# Comparative Nutrient Profiling of Retail Goat and Cow Milk

**DOI:** 10.3390/nu11102282

**Published:** 2019-09-24

**Authors:** Sokratis Stergiadis, Natalja P. Nørskov, Stig Purup, Ian Givens, Michael R. F. Lee

**Affiliations:** 1 Department of Animal Sciences, University of Reading, Agriculture Building, P.O. Box 237, Earley Gate, Reading RG6 6AR, UK; 2 Department of Animal Science, Aarhus University, AU-Foulum, Blichers Alle 20, P.O. Box 50, DK-8830 Tjele, Denmark; natalja.norskov@anis.au.dk (N.P.N.); stig.purup@anis.au.dk (S.P.); 3 Institute for Food Nutrition and Health, University of Reading, Agriculture Building, P.O. Box 237, Earley Gate, Reading RG6 6AR, UK; d.i.givens@reading.ac.uk; 4 Rothamsted Research, North Wyke, Okehampton, Devon EX20 2SB, UK; 5 Bristol Veterinary School, University of Bristol, Langford, Somerset BS40 5DU, UK

**Keywords:** goat milk, fatty acids, minerals, phytoestrogens, retail, dairy, management

## Abstract

Goat milk is globally consumed but nutritional profiling at retail level is scarce. This study compared the nutrient composition of retail cow and goat milk (basic solids, fatty acids, minerals, and phytoestrogens) throughout the year and quantified the potential implications on the consumers’ nutrient intakes. When compared to cow milk, goat milk demonstrated nutritionally desirable traits, such as lower concentrations of C12:0, C14:0, C16:0 and Na: K ratio, and the higher concentrations of *cis* polyunsaturated fatty acids (PUFA), eicosapentaenoic acid (EPA), docosahexaenoic acid (DHA), isoflavones, B, Cu, Mg, Mn, P and I, although the latter may be less desirable in cases of high milk intakes. However, in contrast with nutritional targets, it had lower concentrations of omega-3 PUFA, vaccenic acid, lignans, Ca, S and Zn. The extent of these differences was strongly influenced by season and may demonstrate a combination of differences on intrinsic species metabolism, and farm breeding/husbandry practices.

## 1. Introduction

Dairy goats have been traditionally used for milk production across the globe, and in particular Asia, Africa and Europe, which produce 58.4%, 24.1% and 14.2% of world’s goat milk, respectively [1]. As dairy goat production systems thrive within arid and semi-arid environments such as Asia and Africa, global dairy goat production mainly represents extensive production systems with milk being self-consumed [2]. However, a smaller proportion of global goat milk, mainly produced in Europe and Latin America, is commoditized to dairy products [2]. Although dairy goat farming is traditionally practiced in Southern Europe, including production systems of variant intensity [1,2], dairy goat farming in the UK has increased over the last 25 years [3]. In the UK, there are 40,000–45,000 goats, yielding nearly 34 million litres of milk annually, thus being 0.2% of the volume of the UK’s cow milk production [3]. Although the majority of UK goat milk is used for the production of butter, cheese and yoghurt, liquid retail milk from various brands is also available [3].

Potential health benefits from the consumption of goat milk were recently reviewed, including hypoallergenicity, and improvements in gastro-intestinal disorders, Fe and Cu absorption, growth rates, bone density, and blood levels of vitamin A, Ca, thiamine, riboflavin, niacin, and cholesterol, however, claims around human health still mostly rely on anecdotal evidence, which is also used in industry promotional material and within the media [4,5]. Given that the effect of species, breeds, husbandry practices, and season strongly influences the nutritional quality of milk [6,7,8,9,10], differences between cow and goat milk are expected, while their extent may also differ between and within countries. However, there is a lack of detailed nutrient profiling of retail goat milk in most countries, including the UK. The aim of this study was therefore to (i) investigate the differences in the nutritional profiles (basic solids composition, fatty acids (FA), minerals and phytoestrogens) between cow and goat retail milk, (ii) assess the seasonal effect on the observed differences, and (iii) quantify the potential implications on the consumers’ nutrient intakes.

The discussion in the present work focuses on milk FA profiles, minerals and phytoestrogens. Milk and dairy products are the main source of saturated FA (SFA) in human nutrition, also including those deemed responsible for increased risk of cardiovascular disease (C12:0, C14:0, and C16:0) [11,12]. Total SFA consumption is currently higher than the recommended levels and nutritional recommendations ask for a reduction in their consumption (to contribute less than 10% of total energy intake [11,12]). However, milk also contains several monounsaturated FA (MUFA) and polyunsaturated FA (PUFA) which have been associated with beneficial effects on human health [13,14,15,16,17]. The main beneficial MUFA in milk are c9 C18:1 (oleic acid; OA) and t11 C18:1 (vaccenic acid; VA), while the main beneficial PUFA include c9t11 C18:2 (rumenic acid; RA), and the omega-3 (*n*-3) c9c12c15 C18:3 (α-linolenic acid; ALNA), c5c8c11c14c17 C20:5 (eicosapentaenoic acid; EPA), c7c10c13c16c19 C22:5 (docosapentaenoic acid; DPA) and c4c7c10c13c16c19 C22:6 (docosahexaenoic acid; DHA). Minerals are essential to the human body and play numerous vital roles, including (but not restricted to) enzyme co-factor activity, metallo-proteins, vitamin and bone formation, osmolarity, nutrient absorption and oxygen transport, as previously presented in multiple books and publications [18]. Milk is a good source of the macro minerals Ca, Mg, P and K as well as three micro minerals I, Se and Zn [19,20]. It also contains the macro minerals Na and S along with the micro minerals B, Co, Cu, Fe, Mn, Mo and Ni, although it is not considered a major source of these minerals in human diets [10,19,21]. Phytoestrogens (including lignans, isoflavones and coumestans), and in particular equol, have been associated with health benefits, such as reduced risk for cardiovascular disease, type-2 diabetes, certain cancers, as well as symptoms of osteoporosis, metabolic syndrome and menopause [22,23,24,25,26]. However, in contrast with FA and minerals, research on the potential effect of phytoestrogen consumption does not suffice to generate nutritional recommendations and therefore, there are no available reference intake levels [25].

## 2. Materials and Methods

### 2.1. Experimental Design

Milk samples (*n* = 84) from four brands of conventional retail cow milk (*n* = 48) and three brands of conventional retail goat milk (*n* = 36) were collected at the second week of each month between March 2016 and February 2017. Retail outlets (supermarkets) were located within an 8 km radius from the University of Reading. Cow milk brands were collected from the four supermarkets with the highest market share in the UK while goat milk brands were the three brands available in the survey area during the study. All samples were whole, pasteurized and homogenized milk. The commercial containers (typically made of high-density polyethylene or carton) with the furthest “best before” date, at the day of collection, were sampled, aiming to represent the bottles with the freshest milk available. The commercial containers were immediately transferred in cool-boxes to the laboratories of the University of Reading, where they were aliquoted in 30 mL sterile, screw-top, polypropylene containers and stored at −20 °C. Milk composition is influenced by several factors, the most influential of which are considered to be the animal species, breed and diet [8,27,28,29]. The present study has collected milk at retail and therefore background information beyond species is not known. However, the conventional dairy production in the UK, which represents the majority of the UK dairy farms, is typically using Holstein dairy cows [8,30]. Cows are housed indoors between approximately October and March, fed diets based on conserved forage (mainly grass and maize silage) and concentrate feeds [8,30]. Between April and September, cows are turned out to pasture, and grazing intake may contribute different amounts towards the total feed intake, depending on the farm’s management intensity [8,31]. In contrast, goat-farming systems in the UK are more intensive in terms of diets and housing [32,33]. Notably, although 95% of the UK dairy cow farms graze cows during the grazing season, this practice is only used in 17% of the UK dairy goat farms [30,33]. Goats are housed all year round and without access to pasture, although access to exercise yards may be provided [32,33]. Their diet is more constant throughout the year comprising mainly of a pelleted concentrate feed with access to conserved forage [32,33]. Therefore, the differences found between goat and cow milk composition may be explained by intrinsic species metabolic differences, or the corresponding husbandry practices, or by the combination of both, and these are explained in the Discussion section for each milk composition parameter.

### 2.2. Milk Analysis

Milk was processed by aliquoting each collected milk sample into four 30 mL containers for separate analysis at multiple laboratories, prior to freezing. The first set of 30 mL aliquots was sent as fresh milk (not frozen) for basic composition analysis to National Milk Laboratories (Wolverhampton, UK). Milk concentrations of fat, protein, casein and lactose were measured by Fourier Transform InfraRed spectroscopy (MilkoScanTM 7RM; FOSS, Denmark), while somatic cell count (SCC) were measured by flow cytometry (FossomaticTM 7; FOSS, Denmark). The second, frozen, 30 mL milk sample was thawed overnight at 0–4 °C for FA profiling. The combination of techniques used to analyse milk FA profile were previously reported [7]. The third 30 mL milk samples were submitted for commercial mineral analysis, excluding iodine, to NUvetNA (University of Nottingham, Sutton Bonnigton, UK) using a next day delivery service and enclosed in polystyrene boxes containing ice packs, so that they remain frozen during transport. For iodine analysis, milk was defatted by centrifugation and then a tetramethylammonium hydroxide extraction was carried out before analysis by Inductively Coupled Plasma Mass Spectrometry (ICP-MS). For all other minerals, the sample were microwave acid-digested with nitric acid and hydrogen peroxide and analysed by ICP-MS. Every run had internal quality control and appropriate certified reference materials. The final, frozen, 30 mL milk sample was posted to Aarhus University, Denmark, following the same packaging, postage and transport approaches as described above. Upon delivery, samples were immediately stored at −20 °C until analysis for phytoestrogen concentrations. The analysis of phytoestrogens was performed according to Nørskov et al. [34].

### 2.3. Statistical Analysis

Analysis of variance (ANOVA) was performed by linear mixed effects models and the residual maximum likelihood analysis algorithm in GenStat 17th Edition (VSN International, Hemel Hempstead, UK; [35]). Species (cow, goat), month (March 2016–February 2017), and their interaction, were used as fixed factors while milk ID (the unique brand for each of the four cow and three goat milks) was used as random factor. The effect of the main treatments and their interaction was considered significant at *p* < 0.05, while tendencies were considered at 0.05 < *p* < 0.10. The residual diagnostics relied on normality plots and data did not deviate from normality, except for SCC, which required logarithmic transformation prior to analysis. Pairwise comparison of means (*p* < 0.05) was carried out when the effect of the species × month interaction was significant, using Fisher’s Least Significant Difference test. Desaturase activity indices were calculated as previously described [36].

### 2.4. Modelling of Nutrient Intakes by Consumers

In order to model the impact of switching from cow to goat milk consumption, the present study relied on the average milk and dairy fat intakes reported in the National Diet and Nutrition Survey (NDNS) rolling programme, from Years 1–4 combined [37]. These data, combined with the nutrient composition data generated in the present study, were used to calculate the nutrient intakes from cow and goat milk, assuming, where necessary, the FA content of milk fat to be 93.3% [38]. These nutrient intakes were then compared against (i) nutritional recommendations from the data tables of the NDNS results from years 7 and 8 for Ca, Cu, I, Mg, P, Na and Zn [39], and (ii) nutritional recommendations for all individual/group fatty acid intakes from the draft report of the UK’s Scientific Advisory Committee on Nutrition [40], except for the recommendations for the sum of C12:0 + C14:0 + C16:0 (taken from the French Food Safety Agency [41]), and the recommendation for the sum of EPA + DHA (taken from the European Food Safety Authority [11]). When recommendations were presented as % of energy intake, the energy content of fat (at 37 kJ/g) and dietary reference values for energy intake for different genders and age groups were taken from, or calculated according to, the UK’s Scientific Advisory Committee on Nutrition [42]. Atherogenicity and thrombogenicity indices were calculated as previously described [43].

## 3. Results

### 3.1. Basic Composition

The effect of species was significant on the concentrations of protein, casein, lactose, and SCC (Table 1). When compared with cow milk, goat milk had lower concentrations of protein (−3.1%), casein (−15.7%), and lactose (−8.7%) but higher contents of SCC (+386.7%).

**Figure 1:**
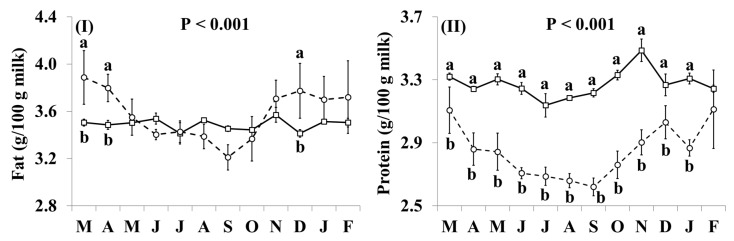

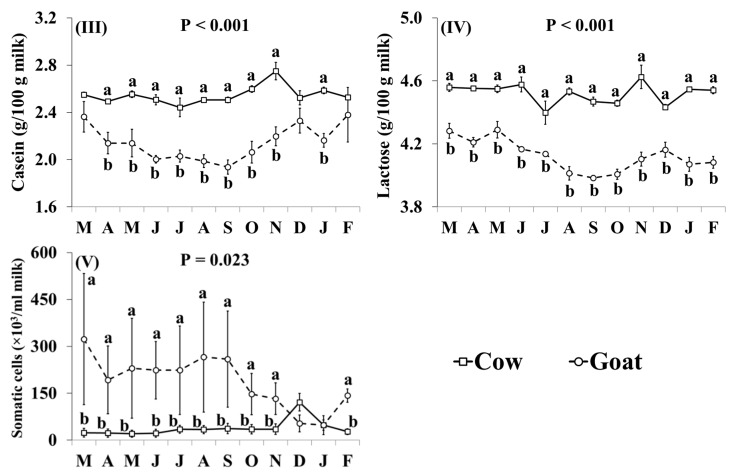
Interaction means ± SE (error bars) for the effects of species (cow, goat) and month (in order of appearance from left to right in Axis X: M, March; A, April; M, May; J, June; J, July; A, August; S, September; O, October; N, November; D, December; J, January; F, February) on the basic composition of retail milk: (**I**) fat content; (**II**) protein content; (**III**) casein content; (**IV**) lactose content; (**V**) somatic cell count. P represents the ANOVA *p*-value for the interaction. Means for species and within a month with different lowercase letters are significantly different according to Fisher’s Least Significant Difference test (*p* < 0.05).

In addition, the effect of the species × month interaction was significant for all basic composition parameters (Figure 1). Goat milk contained more fat than cow milk in March, April and December, but there was no significant difference during the remaining months (Figure 1I). The concentrations of protein, casein and lactose (Figure 1II–IV, respectively) were lower in goat milk than in cow milk throughout the year, but the differences were not statistically significant in February for protein, and in March, December and February for casein. The SCC content of goat milk was higher throughout the year, except for December and January when the difference was not statistically significant, while the relative difference in the SCC content was lower between October and February (Figure 1V).

### 3.2. Fatty Acid Profile

The effect of species was significant on the concentrations of C12:0, C14:0, C16:0, c9c12 C18:2 (linoleic acid; LA), EPA, DHA, SFA, *trans* MUFA, *cis* PUFA, *trans* PUFA, *cis*/*trans* + *trans*/*cis* PUFA, *n*-3, omega-6 (*n*-6), and *trans* FA, as well as for the ratio *n*-3/*n*-6 and the desaturase activity indices C14:1/C14:0, C16:1/C16:0 and RA/VA (Table 2). In comparison with cow milk, goat milk had higher concentrations of C12:0 (+24.8%), LA (+52.8%), DHA (+123.8%), SFA (+2.2%), *cis* PUFA (+29.5%), *n*-6 (+40.7%) and RA/VA ratio. In contrast, it had lower concentrations of C14:0 (−6.8%), C16:0 (−8.5%), VA (−41.2%), EPA (−26.8%), *trans* MUFA (−20.9%), *trans* PUFA (−82.6%), *cis*/*trans* + *trans*/*cis* PUFA (−24.0%), *n*-3 (−32.2%), *trans* FA (−21.6%), and ratios of *n*-3/*n*-6, C14:1/C14:0, and C16:1/C16:0 (Table 2). The detailed FA profile of cow and goat milk is presented in the Supplementary Materials (Table S1).

**Figure 2:**
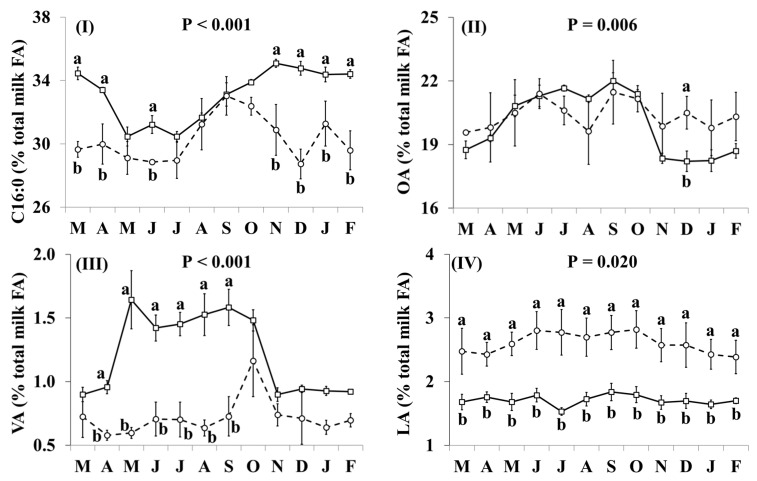

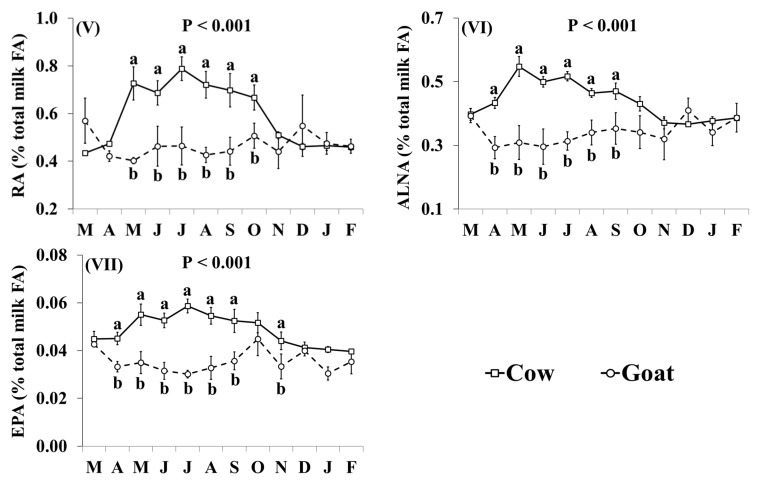
Interaction means ± SE (error bars) for the effects of species (cow, goat) and month (in order of appearance from left to right in Axis X: M, March; A, April; M, May; J, June; J, July; A, August; S, September; O, October; N, November; D, December; J, January; F, February) on the individual fatty acid profile of retail milk: (**I**) C16:0, palmitic acid; (**II**) OA, oleic acid; (**III**) VA, vaccenic acid; (**IV**) LA, linoleic acid; (**V**) RA, rumenic acid; (**VI**) ALNA, α-linolenic acid; (**VII**) EPA, eicosapentaenoic acid. P represents the ANOVA *p*-value for the interaction. Means for species and within a month with different lowercase letters are significantly different according to Fisher’s Least Significant Difference test (*p* < 0.05).

**Figure 3:**
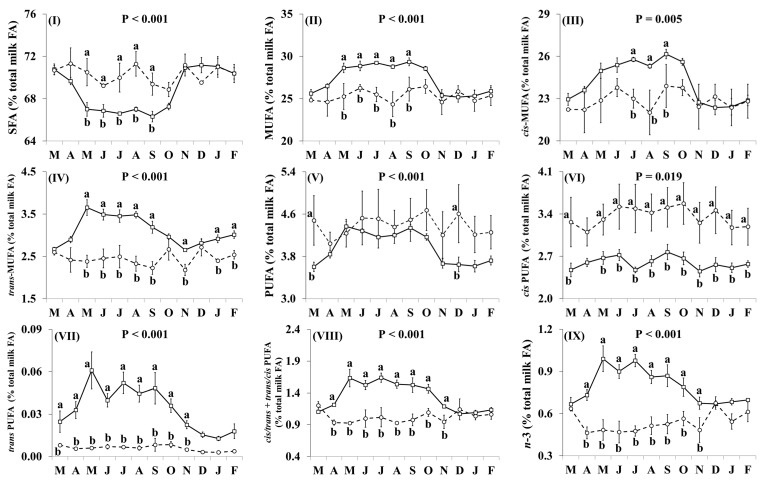

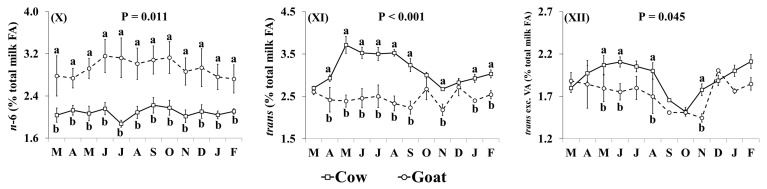
Interaction means ± SE (error bars) for the effects of species (cow, goat) and month (in order of appearance from left to right in Axis X: M, March; A, April; M, May; J, June; J, July; A, August; S, September; O, October; N, November; D, December; J, January; F, February) on the fatty acid (FA) group profile of retail milk: (**I**) SFA, saturated FA; (**II**) MUFA, monounsaturated FA; (**III**) *cis*-MUFA, (**IV**) *trans*-MUFA, (**V**) PUFA, polyunsaturated FA; (**VI**) *cis*-PUFA, (**VII**) *trans*-PUFA, (**VIII**) *cis/trans* + *trans/cis* PUFA, (**IX**) *n*-3, omega-3 PUFA; (**X**) *n*-6, omega-6 PUFA; (**XI**) total *trans* FA, (**XII**) total *trans* FA, excluding VA (vaccenic acid). P represents the ANOVA *p*-value for the interaction. Means for species and within a month with different lowercase letters are significantly different according to Fisher’s Least Significant Difference test (*p* < 0.05).

In addition, the effect of the species × month interaction was significant for many of the individual FA, including C16:0, OA, VA, LA, RA, ALNA, EPA (Figure 2) and FA groups, including SFA, MUFA, *cis* MUFA, *trans* MUFA, PUFA, *cis* PUFA, *trans* PUFA, *cis/trans* + *trans/cis* PUFA, *n*-3, *n*-6, *trans*, and *trans* excluding VA (Figure 3), as well as the ratio *n*-3/*n*-6 and the indices of atherogenicity, thrombogenicity (Supplementary Materials, Figure S1) and desaturase activity (Supplementary Materials, Figure S2). Goat milk contained less C16:0 than cow milk during March, April, June, and November–February (Figure 2I). Concentrations of OA in goat milk were higher in December but there was no significant difference in any other month (Figure 2II). When compared with cow milk, goat milk had higher concentrations of SFA (Figure 3I) and lower concentrations of VA (Figure 2III), RA (Figure 2V), ALNA (Figure 2VI), EPA (Figure VII), MUFA (Figure 3II), and *n*-3 (Figure IX) typically between April and October, although some of the differences at the beginning and end of this period were not statistically significant. For EPA and *n*-3, the difference was also significant in November. Concentrations of LA (Figure 2IV), *cis* PUFA (Figure 3VI) and *n*-6 (Figure 3X) were higher in goat milk throughout the year, with the only non-significant difference appearing for *cis* PUFA in April. Goat milk had lower concentrations of *cis* MUFA than cow milk between July and September (Figure 3III) and of PUFA in March and December (Figure 3V). When compared with cow milk, goat milk had lower concentrations of *trans* MUFA (Figure 3IV) and *trans* FA (Figure 3XI) but differences were not statistically significant in March and October. *Trans* PUFA (Figure 3VII) and *cis/trans* + *trans/cis* PUFA (Figure 3VIII) were found in lower concentrations in goat milk in April–November, while for *trans* PUFA this difference also extended to March. Concentrations of *trans* FA, when excluding VA, were lower in goat milk than cow milk in May, June, August and November (Figure 3XII).

### 3.3. Mineral Concentrations

The effect of species was significant on the concentrations of B, Ca, Cu, I, K, Mg, Mn, Na, P, S, and Zn in milk (Table 3). When compared with cow milk, goat milk had higher concentrations of B (+49.3%), Cu (+102.8%), I (+85.6%), K (+33.3%), Mg (+27.3%), Mn (+144.9%), and P (+8.6%), and lower concentrations of Ca (–5.5%), Na (–6.0%), S (–9.1%), and Zn (–15.4%) (Table 3).

**Figure 4:**
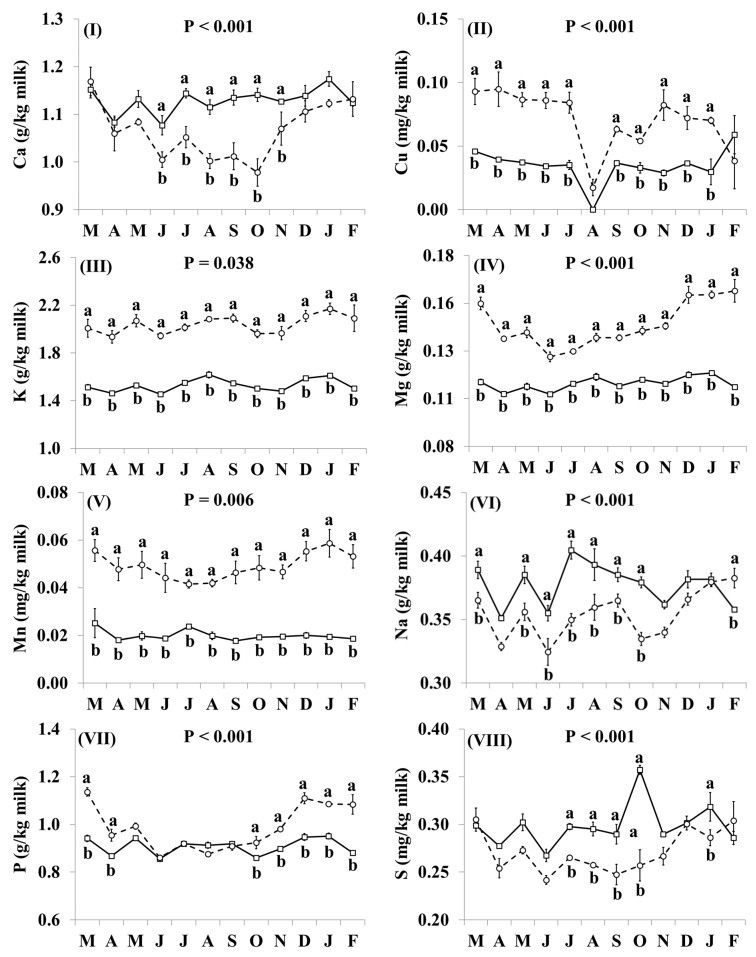

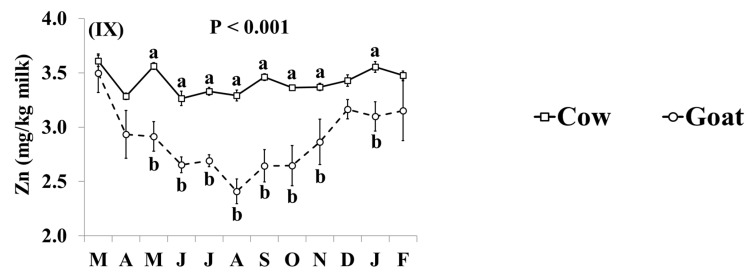
Interaction means ± SE (error bars) for the effects of species (cow, goat) and month (in order of appearance from left to right in Axis X: M, March; A, April; M, May; J, June; J, July; A, August; S, September; O, October; N, November; D, December; J, January; F, February) on mineral concentrations of retail milk: (**I**) Ca, calcium, (**II**) Cu, copper, (**III**) K, potassium, (**IV**) Mg, magnesium, (**V**) Mn, manganese, (**VI**) Na, sodium, (**VII**) P, phosphorus, (**VIII**) S, sulphur, (**IX**) Zn, zinc. P represents the ANOVA *p*-value for the interaction. Means for species and within a month with different lowercase letters are significantly different according to Fisher’s Least Significant Difference test (*p* < 0.05).

In addition, the effect of the species × month interaction was significant for Ca, Cu, K, Mg, Mn, Na, P, S, and Zn (Figure 4). Goat milk had lower concentrations of Ca than cow milk between June and November (Figure 4I). Goat milk had higher concentrations of Cu throughout the year, except for August and December when there was no significant difference (Figure 4II). The concentrations of K, Mg and Mn were higher in goat milk than in cow milk throughout the year with the relative difference being highest between December and February (Figure 4III–V, respectively). Concentrations of Na were lower in goat milk in March, May–October, and February (Figure 4VI). Goat milk also had higher concentrations of P than cow milk between October and April, but not between May and September (Figure 4VII). When compared with cow milk, goat milk had lower concentrations of S in July–October and January (Figure 4VIII), and lower concentrations of Zn in May–November and January (Figure 4IX).

### 3.4. Phytoestrogen Concentrations

The effect of species was significant on the concentrations of secoisolariciresinol, matairesinol, lariciresinol, hydroxymatairesinol, enterolactone, daidzein, glycitein, naringenin, equol, plant lignans, mammalian lignans, and total lignans (Table 4). When compared with cow milk, goat milk had lower concentrations of secoisolariciresinol (−62.7%), matairesinol (−50.0%), lariciresinol (−47.4%), hydroxymatairesinol (−56.2%), enterolactone (−67.6%), plant lignans (−52.5%), mammalian lignans (−66.6%) and total lignans (−66.5%), and higher concentrations of daidzein (+747.3%), glycitein (+167.5%), naringenin (+185%), equol (+985.8%) and isoflavones (+964.3%) (Table 4).

**Figure 5:**
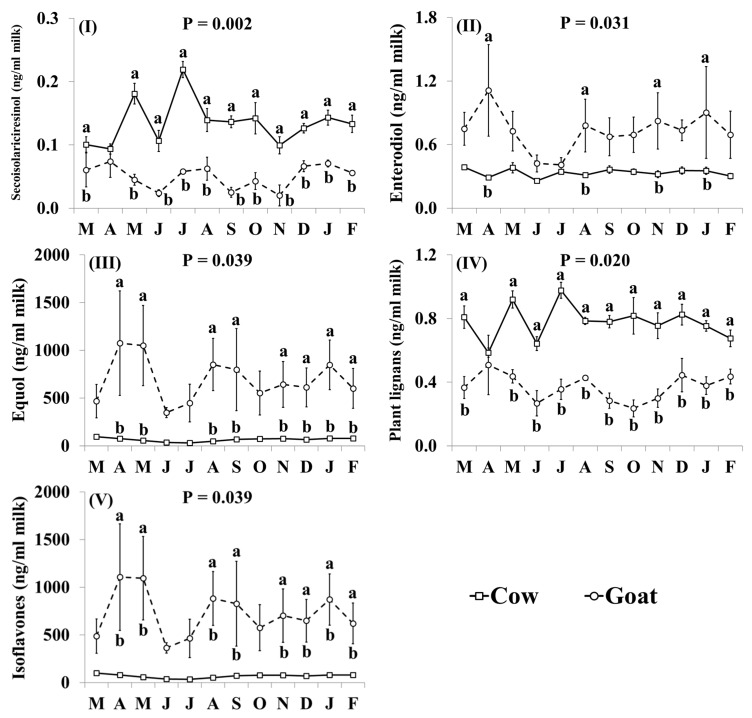
Interaction means ± SE (error bars) for the effects of species (cow, goat) and month (in order of appearance from left to right in Axis X: M, March; A, April; M, May; J, June; J, July; A, August; S, September; O, October; N, November; D, December; J, January; F, February) on phytoestrogen concentrations of retail milk: (**I**) secosisolariciresinol, (**II**) enterodiol, (**III**) equol, (**IV**) total plant lignans, (**V**) total isoflavones. P represents the ANOVA *p*-value for the interaction. Means for species and within a month with different lowercase letters are significantly different according to Fisher’s Least Significant Difference test (*p* < 0.05).

In addition, the effect of the species × month interaction was significant for secoisolariciresinol, enterodiol, equol, plant lignans and isoflavones (Figure 5). Concentrations of secoisolariciresinol (Figure 5I) and plant lignans (Figure 5IV) were lower in goat milk, than in cow milk, throughout the year although the difference was smallest and not statistically significant, in April. Goat milk had lower concentrations of enterodiol than cow milk in April, August, November and January (Figure 5II). Goat milk had higher concentrations of equol (Figure 5III) and isoflavones (Figure 5V) than cow milk throughout the year, although differences were not statistically significant in June and July. 

## 4. Discussion

### 4.1. Basic Composition of Retail Goat Milk

The similar fat content between goat and cow milk is partly in line with previous work [44] but also in contrast with other studies [45,46]. However, fat standardisation is a common practice in the conventional cow milk supply chains in the UK and its actual fat content is therefore modified prior to reaching the retail outlets. The fact that goat milk contained less protein and casein than cow milk is in contrast with a number of studies [27,44,46] although differences are not always statistically significant [45]. As animal genetics are a strong determinant of protein content in goats and cows [8,9,28,47], it is possible that contradictory results between studies may partly originate on the diverse genetic background of the goats and cows used across the different studies and countries. In addition, the relatively low dietary forage:concentrate ratio in the UK dairy herds increases milk yield but may reflect the lower proportion of milk solids. The lower lactose concentrations of goat milk have previously been reported [46] although in other studies [44,45] there was no significant difference. In dairy cows, a negative correlation has been previously reported between contents of lactose and SCC [48], the latter were almost five times (numerically) higher in goat milk (although the difference was not statistically significant; *p* = 0.053) and this may explain the lower lactose concentrations in goat milk in the present work.

### 4.2. Fatty Acid Profile of Retail Goat Milk

Although previous studies reported similar SFA concentrations between goat and cow milk [49,50], the present work found that goat milk contained more SFA. However, goat milk contained less C14:0 and C16:0 and more of the shorter chain SFA (with 6–12 atoms of carbon). These differences in individual SFA between goat and cow milk are consistent across many studies [44,45,49,50]. Recent work has highlighted differences in the mRNA abundance of 9 genes, including *FASN* (which encodes the FA synthase to catalyse FA synthesis), which demonstrate a higher de novo synthesis of fat in goats than in cows [51]. The activity of FA synthase in goats was ten times higher than in cows in other studies [52]. Given that the de novo FA synthesis in the mammary gland is responsible for the production of SFA with 4–14 atoms of carbon (and approximately 50% of C16:0) in milk [6,44], it is expected that animals with higher mammary lipogenesis would produce milk richer in these FA. Furthermore, cows are more efficient than goats in transferring C16:0 from the blood to the mammary gland (following absorption) and this explains the consistently higher C16:0 concentrations in cow milk across all studies [44,45,49,50]. In the present work, this difference has disappeared during the typical UK grazing season, this is probably because (i) grazing is known to reduce milk C16:0 concentrations [8,25,29,31,53], and (ii) grazing is a common feeding practice in the UK dairy cow systems, but not necessarily in the year-round housed intensive dairy goat systems [8,31,33]. As a result, cow milk contained less SFA than goat milk during the grazing season.

Goat milk has been previously reported to contain less OA under control diets [45], contain less OA only after dietary oil supplementation [44] or having similar concentrations to cow milk [49,50]. The latter has also been observed in the present study, although only in December goat milk had lower concentrations of OA. In contrast to previous work, which showed similar concentrations of VA between goat and cow milk [44,49,52], in the present study VA concentrations were higher in cow milk. However, the species × month interaction showed that this difference was only observed in summer. Given that milk VA concentrations are primarily driven by pasture intake [8,29,31], this is probably a consequence of the higher pasture contribution to cow diets during the grazing season. Although milk concentrations of *trans* MUFA and total *trans* FA were also higher in cow milk (mainly during the grazing season), this is down to VA’s contribution to these two parameters (around 39%). When VA was excluded from the sum of total *trans* FA, there was no significant overall effect of species, although cow milk still contained more *trans* during the grazing season, potentially because of the higher intakes of ALNA from pasture and its subsequent rumen biohydrogenation to *trans* FA [6,53]. 

The higher LA concentrations in goat milk in the current study are in line with Fougere et al. [45] and Poti et al. [50], but in contrast with Yang et al. [49] and Toral et al. [44], who showed lower concentrations of LA in goat milk. This finding is potentially due to the higher contribution of concentrate feed, which is a rich source of LA and positively correlated with milk LA concentrations [6,8,31] in the intensive dairy goat diets. Although it is expected that concentrate feeds are provided to nearly all cow and goat herds across UK, 38% of goat farms are supplying concentrate feed ad libitum [33], a practice which is not used in the dairy cow herds and is expected to increase concentrate intakes due to their high palatability. In addition, cow milk had higher concentrations of RA, ALNA, EPA and *n*-3 during the grazing season. This is expected because the higher grazing intake of cows during the grazing season will increase the supply of *n*-3, and in particular ALNA [53]. Increased ALNA intakes may increase: (i) ALNA absorption and transfer to the mammary gland and milk, (ii) production of VA in the rumen and delivery to the mammary gland for RA synthesis under the effect of Δ^9^-desaturase, and (iii) the production of EPA under the effect of Δ^6^-desaturase [6,54]. 

The higher concentrations of DHA in goat milk are in contrast with previous work, which showed either no significant effect of species, or higher concentrations of DHA in cow milk and/or higher DHA transfer efficiencies from diet to milk when DHA-rich diets were fed [44,45]. Potential differences in dietary supply of DHA between cow and goat dairy herds might be responsible for this finding in the present work, but this cannot be assessed because data on concentrate supplements used in the different supply chains are not available. 

The lower ratios of C14:1/C14:0 and C16:1/C16:0, the higher ratios of RA/VA and the similar ratio of OA/C18:0 are in line with previous work [44,45]. These findings further support the hypothesis that Δ^9^-desaturase in the mammary gland of goats may have a higher affinity for VA, rather than for C14:0 and C16:0 [6,44]. 

### 4.3. Mineral Concentrations of Retail Goat Milk

The major milk cations are associated within casein micelles, accounting for 70% Ca and 35% Mg, whilst 50% inorganic phosphate (P) are also found within the milk solid fraction [19,55]. Milk S is predominately found within sulpho-amino acids of milk proteins. Not surprisingly, therefore, a positive correlation between milk protein concentration and Ca, Mg, P and S was previously shown for cow milk [20]. In the current study for goat milk, Ca and S were positively correlated with milk protein, whereas the inverse was true for Mg and P (data not shown). This may relate to the closer affinity of Ca and S to protein and the higher proportion of Mg and P contained within the soluble fraction of milk, which are essentially diffusible and therefore more easily influenced by dietary intake. Dunshea et al. [20] reported that Ca, Mg and P in milk were all significantly influenced by dietary supplementation of either concentrates, via increased milk protein content, or minerals, via diffusion into milk. When comparing goat versus cow milk for these three macro-minerals, the current study found lower Ca, and higher concentrations of P and Mg, whereas other work reported higher concentrations of Ca for goat milk [21,56]. Furthermore, the ratio of Ca/P within the current study for UK goats’ milk (1.08) is lower than the reported average (1.20) [57], with previous studies reporting ratios as high as 1.34 and 1.44 from indigenous Italian and Portuguese breeds, respectively [10,58]. These lower concentrations of Ca, but also S, in goat milk in the current study likely relate to the association with milk protein, which was also lower in goat milk. In the previous comparative studies, showing higher concentrations of Ca in goat milk, the protein content in goat milk was also higher than that in the current study [33]. Milk Ca concentrations in the current study were lower than those reported for Greek and Portuguese goat milk [58,59] whereas P and Mg were comparable, thus further emphasizing the association with milk protein. Temporal differences of Ca and P were previously reported, but with no significant change in Mg from a single Greek goat herd, with differences linked to stage of lactation and nutrition [59]. For Italian goat breeds, similar values for Ca were found as in the current UK study, but with lower levels of P and Mg, which also varied significantly across breeds [10]. Although the current study cannot reflect changes within a single herd or across breeds, as the milk represents many herds which may be at different stages of lactation, the temporal variation for Ca and P was higher than that for Mg, and higher for goat than cow milk. This may reflect the similarity of husbandry practices (including calving patterns and diet) in UK dairy goat production (highly intensive), compared to more diverse dairy cow production systems within the UK, and its subsequent influence on milk minerals.

K and Na are the main solely diffusible (liquid milk) cation macro minerals in milk and they exist mainly as free ions, with the rest being associated with citrate, inorganic phosphate and chloride [19]. The concentrations found in the current study are similar to previous work [5,10,21,56,60], but in contrast with other studies [59,61], which reported higher concentrations for Na and lower for K within goat milk. This resulted in the low ratio of Na/K in the current study of 0.18 compared to 0.23, 0.25 and 0.80 reported by Park et al. [21], Trancoso et al. [58] and Kondyli et al. [59], respectively. These differences directly relate to dietary intake with access to pasture, type of concentrates and salt licks/mineral supplements significantly influencing Na and K levels in goat milk [58,59,62]. In contrast within cow milk, Dunshea et al. [20] reported no influence of mineral supplements, concentrates, or conserved forage on either Na or K, in agreement with our cow temporal findings. However, they did show a clear response with access to pasture increasing K by 2 ± 0.8 mg/kg for every extra kg DM consumed.

Se, I, and Zn [18] are all predominately found within the liquid diffusible portion of milk, with some Se also found within milk protein as seleno-amino acids [19]. The concentration of Se reported here and that of Park et al. [21] were broadly similar with 0.013 and 0.010 mg Se/kg for goat and cow, respectively. However, for Zn the current study reported concentrations of approximately 1.6 and 1.8 times lower for cow and goat milk respectively, when compared with Park et al. [21]. Whereas, Trancoso et al. [58] and Kondyli et al. [59] compared to the current study reported similar concentrations of Zn in goat milk, of approximately 3.70 and 2.82 mg/kg, respectively. Our current assessment showed contrasting results, with Se being similar in milk from the two species, whereas Zn was higher in cow milk. In cow milk, Se was shown to be highly correlated with concentrate and mineral intake, whereas Zn showed little response to supplementation [20]. An improved Se transfer into milk has been reported with supplementation of organic sources, such as selenised yeast, through provision of selenomethionine [63]. Although, other supplementation approaches, such as the recently reported nano-Se, may also provide valuable sources for incorporation into product, especially on conserved forage diets [64]. It is therefore highly likely that concentrations of Se and Zn reflect the amounts and type of mineral supplements provided but these data are not available in the current retail survey. Similar to the cow milk study of Dunshea et al. [20], the goat study of Trancoso et al. [58] reported no significant fluctuation in Zn concentrations associated with geographical region. However, Kondyli et al. [59] reported a significant seasonal effect for Zn milk concentration, which, as in the current work, may be associated with stage of lactation [10] or/and mineral supplementation status. Curro et al. [10] also reported significant variation in milk Zn across goat breeds in Italy ranging from 2.29 to 3.19 mg/kg.

Few studies report I in goat milk with the concentrations reported by Park et al. [21] for both goats and cows lower than in our current study by 3 and 1.7 times, respectively. In another recent study [65], goat milk collected from commercial farms in Italy had 14.5% lower concentrations of I (0.575 mg/kg) than retail goat milk in the present work. Previous work has reported that I in cow milk was 50% lower in summer than winter [66]. Milk I status is related to dietary intake, and as most I would be provided in supplementary feeds and mineral supplements, this would explain the lower concentrations in the summer, when the contribution of fresh grass to cow diets is increased. This also agrees with our current findings as most UK dairy goat herds are confined and fed on indoor mixed rations with supplementary minerals and feed [33].

For the remaining micro minerals, B, Cu and Mn were higher in goat than cow milk, whereas no difference was found for Co, Fe, Mo and Ni. Previous work [21,56] reported no difference between cow and goat milk for Fe but at over twice the concentration reported here (0.5–0.8 mg Fe/kg). Whereas, Curro et al. [10] reported comparable levels of Fe within Italian goat milk (0.31 mg Fe/kg), as reported here for UK goat milk, levels of B were considerably higher (1.42 mg B/kg), which may reflect the basal diet. For Cu and Mn, in terms of trend towards higher levels in goat milk and total milk concentration values for both species, the current study agrees with previous comparative studies [21]. For goat milk, seasonal differences were previously shown for Cu and Mn concentrations but with no difference for Fe [59]. Trancoso et al. [58] reported regional differences for goat milk Mn and Fe concentrations, but comparable levels of Mo, although variation was high within regions which they attributed to the influence of soil and seasonality. The same study also assessed Ni and Co status within goat milk, but levels were below the detection limit of 4.0 and 2.0 μg/kg, respectively. The current study detected Ni and Co in goat milk at 0.8 and 0.3 μg/kg, which agrees with their findings based on detection limits with levels likely related to dietary intake.

Unlike the other potentially toxic heavy metals, Cd and Pb, ruminants have been shown to have a requirement for As at levels between 350 and 500 μg/kg DM, with levels <50 μg/kg DM reducing mineralisation of bone [67]. Trancoso et al. [58] also investigated Cd and Pd within goat milk with concentrations below their detection limit of 0.6 and 7.0 μg/kg, which align with the concentrations recorded in the current study of 0.044 and 0.583 μg/kg respectively, and are likely related to dietary intake.

### 4.4. Phytoestrogen Concentrations of Retail Goat Milk

The concentration of isoflavones, lignans and coumestants in milk from cows and goats is influenced mainly by (i) animal diet, which determines the amounts of plant lignans, isoflavones and coumestants entering the digestive tract, and (ii) rumen microbial activity, which determines the extent of synthesis of mammalian lignans and isoflavones using the dietary phytoestrogens as substrates [68,69,70,71]. Milk phytoestrogens concentrations have been reported more extensively for dairy cows [34], than for dairy goats (only one study in France [70]). 

The concentrations of equol, its precursors formononetin and daidzein, in goat milk are substantially higher than in cow milk, whereas the opposite was seen for enterolactone and its precursors secoisolariciresinol, lariciresinol and matairesinol. These differences may be attributed to contrasting dietary supply and/or microbial composition in the rumen. There are several studies investigating the effect of equol synthesis from rumen bacteria by using dietary isoflavones as precursors [68,72,73]. Yao et al. [73] suggested that daidzein supplementation influences rumen microbial composition. In addition, Kasparovska et al. [68] also reported that dairy cow diets which are enriched with isoflavones influence the abundance of Bacteroidetes, Protebacteria, Firmicutes and Planctomycetes in the rumen. In the absence of similar studies in goats, it is not possible to compare whether the bacterial conversion of plant isoflavones to equol differs between species. However, Wang et al. [72] showed that the main bacterial communities in the rumen of goat were composed of Bacteroidetes, Firmicutes and Proteobacteria, all representing major producers of equol [68]. Higher amounts of equol escaping the rumen would be absorbed in the small intestine and transferred into the mammary gland, and eventually milk [74]. It is therefore not surprising that goat milk contains high amounts of equol. 

The higher concentrations of the dietary origin daidzein, genistein and formononetin in goat milk indicate a higher dietary supply of these in goat diets. Soybean (*Glycine max* L.) and clover (*Trifolium* spp. L.) are known as major sources of these isoflavones in animal diets [75]. Clover pastures and silages are not very common in conventional dairy and goat production systems in the UK, but soybean meal is expected to be a contributor of protein in dairy diets for both species. In general, dietary supply of soybean meal tends to be higher in more intensive production systems in order to meet the higher requirements of high-yielding animals for good quality protein. Given that goat milk contained more daidzein, genistein and formononetin, it would not be surprising if this was an effect of higher soybean meal supplementation in the diet of dairy goats than in the diets of dairy cows, because they represent an overall more intensive production system [30,33]. However, in the absence of background data of the dietary practices for the production of the retail samples in the present study, it is rather difficult to identify the exact origins of these differences. Moreover, there is also high variation in the concentrations of formononetin (0.038–37 ng/mL), and equol (130–2120 ng/ml), which highlights that dietary, husbandry and genetic factors influencing milk phytoestrogen concentrations may largely vary between the different production chains of the main suppliers of goat milk in the UK. The higher concentrations of glycitein and naringenin in goat milk may also be a result of higher intakes of soybean meal, as these are also components in soybean (although minor) [76]. 

Opposite to isoflavones, the concentration of lignans were lower in goat milk than in cow milk. The major source of lignans, such as secoisolariciresinol, matairesinol, lariciresinol and hydroxymatairesinol, are grains such as wheat (*Triticum aestivum*), oat (*Avena sativa*) and barley (*Hordeum vulgare*), which are the main constituents of concentrate diets in the UK. However, the contribution of these concentrates into the diets of dairy cows and goats in the supply chains of the brands that were sampled in the present study is not known. It is possible that a higher contribution of soybean meal in the concentrate part of the diet of the dairy goats may have resulted in lower contribution of these cereals than in dairy cow diets. This potentially lower intake would reduce the amounts of plant lignans transferred in goat milk, as well as the substrate for enterodiol synthesis in the rumen [69]. However, potential genetic effects on this finding cannot be excluded. For example, it is possible that cows are more efficient in producing enterolactone than goats because *Prevotella* spp., the main bacteria responsible for the conversion of plant lignans to mammalian lignans [77], was found in high abundance in the rumen of dairy cows in other studies [78]. However, the concentrations of the other mammalian lignan eneterolactone was not significantly different between cows and goats, and higher in goats in four months throughout the year. Notably, the variation in lignan concentrations of goat milk was much smaller than in isoflavones. This may indicate that the nutritional and genetic influences that affect lignan concentrations are more stable throughout the year, than those influencing the concentrations of isoflavones. 

### 4.5. Impact of Consuming Goat Milk on Nutrient Intakes of UK Consumers

Due to compositional differences identified within our retail evaluation, if a consumer switches from cow to goat milk, this will also influence the intake of fatty acids, minerals and phytoestrogens.

#### 4.5.1. Intakes of Fatty Acids 

A switch from cow milk to goat milk for children (years 1–18) and adults (>19 years) would (i) increase the intakes of nutritionally undesirable [11,12,40] SFA (by +163 and +179 mg/day, respectively), (ii) reduce the intakes of the nutritionally beneficial [13,15,17] *n*-3 PUFA (by −27.6 and −30.3 mg/day, respectively) and VA (by −54.4 and −59.6 mg/day, respectively), and (iii) increase the intakes of the essential, but oversupplied in Western diets [79], LA (by +97.9 and +107.3 mg/day, respectively) and *n*-6 PUFA (+92.4 and +101.4 mg/day, respectively). On the contrary, beneficial changes will also be observed. A switch to goat milk for children (years 1–18) and adults (>19 years) would reduce the intakes of the nutritionally undesirable C12:0, C14:0 and C16:0 (by −301 and −330 mg/day, respectively), and *trans* FA (−72.9 and −79.9 mg/day, respectively) and increase the intakes of nutritionally beneficial *cis* PUFA (by +83.7 and +91.8 mg/day, respectively) and EPA + DHA (by +0.43 and +0.48 mg/day, respectively).

Interestingly, when compared with cow milk, consumption of goat milk would increase the contribution to the upper recommended limit (10% of total energy intake [40]) for SFA (from 33.5% to 34.2%), but would reduce the contribution of milk to the upper recommended limit (8% of total energy intake) for C12:0, C14:0 and C16:0 (from 29.2% to 27.5%). This is in line with FAO recommendations [12] to focus on the reduction of specific SFA (C12:0, C14:0 and C16:0) rather than total SFA. This is observed as goat milk was richer in lower chain SFA, including C6:0 (+11%), C8:0 (+107%), C9:0 (+69%) and C10:0 (+219%), thus aligning with previous studies [44,45,49,50]. Some of these FA have been reported to have beneficial effects on human health, including antiviral activity (C8:0) and delaying the growth of tumours (C10:0) [16]. However, switching to goat milk would reduce the contribution of milk to the recommended intakes (450 mg/day [40]) of the beneficial *n*-3 (from 20.0% to 13.6%). Although a reduction to the contribution of milk to the upper recommended intakes for *trans* FA by consuming goat milk (from 8.3% to 6.5%) may be considered desirable, this has been observed mainly to the reduction of VA, which is a beneficial FA [15]. When VA is not taken into account, *trans* FA content in cow and goat milk are similar. A switch to goat milk would also increase the contribution of milk to the dietary requirements for *cis* PUFA, *n*-6 and EPA + DHA but the extent of these differences is biologically small (from 2.3% to 3.0%, from 1.1% to 1.6% and from 3.9% to 4.1%, respectively).

#### 4.5.2. Intakes of Minerals

A switch from cow milk to goat milk for children (years 1–18) and adults (>19 years) would increase the intakes of Cu (by +6.3 and +5.6 μg/day, respectively), I (by +55.9 and +49.7 μg/day, respectively), Mg (by +5.6 and +5.0 mg/day, respectively), P (by +14.1 and +12.5 mg/day, respectively), K (by +91.8 and +81.6 mg/day, respectively), Mn (by +5.2 and +4.6 μg/day, respectively), and B (by +15.7 and +13.9 μg/day, respectively). These are nutrients highly relevant to human health [18]. Cu enhances haemoglobin and pigments formation and enzyme function, Mg is a major enzyme co-factor and essential for muscle and nerve function, P is essential for acid-base balance, protein and energy metabolism and membrane structure, K is required for nerve conduction, muscle contraction and maintenance of water and acid-base balance, Mn is a catalytic co-factor and activator for a number of enzymes, and B has been for a long time considered only an essential element in plants but recently has been shown to affect many mammalian enzymes, bone development, mineralization and energy metabolism [80].

The most striking difference in the mineral content between cow and goat milk was for I, an essential co-factor in thyroid hormone formation controlling metabolic rate. A switch to goat milk will increase the contribution of milk to I dietary requirements for children (years 1–18; from 62.4% to 115.7%) and adults (>19 years; from 41.6% to 77%). For adults, this is beneficial because I deficiency is among the most widely documented deficiencies globally (including the UK), and the world’s greatest single cause of preventable brain damage, impaired intellectual ability, low IQ scores, and poor school and work performance, with such problems being documented even in populations classified as mildly I deficient [81,82]. An extra supply of I from milk (which is the main source of I in many countries [83]) may have a beneficial impact on consumer I intake and reduce I deficiency prevalence. However, the fine line between meeting the requirements and the upper tolerable limits in children may raise concerns. This is because under the average milk intakes in the UK for children 1–3 years old [37], goat milk would supply an excess of 110 μg/day over the recommended intake (70 μg/day; [37]) and only 20 μg/day lower than the upper tolerable limit for this age (200 μg/day; [84]). Notably, the UK’s National Health Service recommends at least 350 mL of milk a day to young children, but the upper tolerable limit for children aged 1–3 years can be reached by consumption of only 297 mL of goat milk. This issue is resolved in 11–18 years and later life (where milk consumption is reduced and I requirements are increased) and switching to goat milk can increase contribution of I to the recommended intakes (140 μg/day in adults; [37]) from 38.1% to 70.7%, which is considered nutritionally beneficial. A switch to goat milk would also increase the contribution of milk to the dietary requirements for Cu, Mg, P, and K, which can be considered nutritionally beneficial, although the extent of these differences are smaller (from 0.8% to 1.5%, from 10.0% to 12.7%, and from 12.9% to 17.2%, respectively).

A switch from cow milk to goat milk for children (years 1–18) and adults (>19 years) would reduce the intakes of Ca (−11.2 and −9.9 mg/day, respectively), Na (by −4.1 and −3.7 mg/day, respectively), Zn (by −95.1 and −84.5 μg/day, respectively), and S (by −4.3 and −4.3 μg/day, respectively). This switch to goat milk would therefore reduce the contribution of milk to the dietary requirements for Ca, Na and Zn, but the extent of these differences is small (from 32.2% to 30.4%, from 5.8% to 5.4%, and from 8.0% to 6.8%, respectively). These nutrients are also highly relevant to human health [18]. Ca is required for bone development and growth. Na, similar to K, is required for nerve conduction, muscle contraction and maintaining water and acid-base balance. Zn is an essential co-factor for more than 200 enzymes involved in digestion, metabolism reproduction and wound healing. S is a major co-factor and component of sulpho-amino acids and vitamins. However, the concentrations and ratios of Na to K may influence consumer purchase decisions towards buying goat milk, especially for people suffering from high blood pressure or under dialysis, conditions in which a lower Na to K ratio is recommended [85].

Cow and goat milk had similar concentrations of Co (responsible for the formation of vitamin B12 in the rumen), Fe (demonstrating enzyme and protein functions and participating in the formation of haemoglobin), Mo (demonstrating enzyme functions, including xanthine, aldehyde and sulphite oxidases), Ni (having a role in membrane and nucleic acid metabolism and rumen microbial enzyme activation) and Se (being an essential constituent of more than 20 seleno-proteins with a critical role in reproduction, thyroid hormone metabolism, DNA synthesis and protection from oxidative damage and infection) [18]. Therefore, switching between cow and goat milk consumption is not expected to affect the consumer intakes of these nutrients. Cow and goat milk contain negligible amounts of heavy metals because the concentrations found within goat and cow milk in the current study were well below the maximum permissible limit stipulated by the European Commission Regulation directive EC333 for milk e.g., 0.02 mg Pb/kg [86].

#### 4.5.3. Intakes of Phytoestrogens

Despite the high variation in phytoestrogen concentrations among the goat milk samples, a switch from cow milk to goat milk for children (years 1–18) and adults (>19 years) would potentially increase the intakes of daidzein (by +1.2 and +1.1 μg/day, respectively), glycitein (by +0.6 and +0.5 μg/day, respectively), naringenin (by +56.1 and +49.8 ng/day, respectively), and equol (by +109.8 and +97.6 μg/day, respectively). The most striking anticipated effect on isoflavones intakes, by switching to goat milk, was therefore estimated to be the potentially higher intakes of equol, as differences in the other isoflavones were numerically small. Potential health benefits from an increased consumption of equol include decreased risks of breast, prostate and colon cancers, osteoporosis, cardiovascular diseases and hormone-dependent conditions [87]. On the contrary, a switch from cow milk to goat milk for children (years 1–18) and adults (>19 years) would reduce the intakes of secoisolariciresinol (by −54.4 and −59.6 ng/day, respectively), matairesinol (by −10.9 and −9.6 ng/day, respectively), lariciresinol (by −28.6 and −25.4 ng/day, respectively), hydroxymatairesinol (by −17.3 and −15.4 ng/day, respectively) and enterolactone (by −7.3 and −6.5 ng/day, respectively). This can be considered undesirable because enterolactone and its precursors have also been associated with beneficial health effects similar to equol [22].

However, it is not possible to draw any conclusion about the potential effect of the differences between cow and goat milk on the phytoestrogen concentrations. This is partly due to the relatively low numerical differences for most of them (although statistically significant) but, most importantly, because of limited research on their effect on health and the consequent absence of relevant nutritional recommendations [25].

### 4.6. Strengths and Limitations of the Study

This is the first study to present the nutrient composition of goat milk in the UK and model the subsequent effect of nutrient intake in UK consumers, by switching from cow to goat milk. This information can be used by health organisations and nutritionists when public nutritional advice is developed. The impact of goat milk consumption on nutrients intake has been assessed for the different age groups and revealed that characteristics which are considered desirable in adults (e.g., the high I concentrations) may pose an area of concern for children. The present work covered a 12-month period, thus capturing and investigating the seasonal variation in milk composition, a process that revealed that nutritional advice should also account for temporal milk composition changes. The main limitation of the current study is that it relies on milk samples collected solely in the UK. Given that production system influences milk composition, and goat husbandry practices are highly variant across the world, the comparative analysis between cow and goat milk may differ between countries. Therefore, the application of the results in other countries may require further investigation in the future. In addition, in the present study, there was an absence of background information on animal breed and husbandry practices for the produced milk (as this is a retail survey). Although the potential explanations for the observed differences rely on data around husbandry practices from UK studies and producers’ organisations, these speculations should be further investigated in the future with studies at the farm or animal level.

## 5. Conclusions

In comparison to retail cow milk, goat milk showed a number of nutritionally desirable characteristics, including lower concentrations of individual SFA which are associated with increased risk of cardiovascular diseases (C12:0, C14:0, C16:0), an improved Na:K ratio, higher concentrations of fatty acids with positive effect on human health, including cis PUFA, EPA, DHA, and contained isoflavones, B, Cu, Mg, Mn, P. The higher contents of I in goat milk may be desirable in adults (as I deficiency is a main nutrient deficiency at global scale), but care should be taken in children 1–3 years old as consumption of reasonable amounts of goat milk can reach upper tolerable limits of I supply. Furthermore, goat milk had lower concentrations of beneficial omega-3 PUFA, VA, and lignans, and the minerals Ca, S and Zn which play major metabolic roles in the human body. The compositional differences between cow and goat milk may be potentially explained by husbandry practices between cow and goat dairy production systems in the UK, and in particular the lower grazing intake and forage: concentrate ratio and the higher concentrate and mineral supplementation of the dairy goat diets. However, intrinsic metabolic differences between species may have also contributed to this difference. Although a number of compositional differences have been identified which also influence the nutrient intake of consumers, the implication of these differences on human health are not known.

## Figures and Tables

**Table 1:** Means (and average SE) and ANOVA *p*-values for the basic composition of cow and goat retail milk collected throughout the study.

	Cow	Goat		ANOVA
Milk Solids	*n* = 48	*n* = 36	SE	*p*-Values *^a^*
Fat (g/100g milk)	3.49	3.58	0.033	ns
Protein (g/100g milk)	3.27	2.85	0.027	**
Casein (g/100g milk)	2.55	2.14	0.025	**
Lactose (g/100g milk)	4.52	4.13	0.016	***
SCC (somatic cell count) *^b^* (×10^3^/mL milk)	38	187	18.7	†

*^a^* Significances were declared at ***, *p* < 0.001; **, *p* < 0.01; *, *p* < 0.05; †, 0.05 < *p* < 0.10 (trend); ns, *p* > 0.10 (non-significant); *^b^* SCC, somatic cell count.

**Table 2:** Means (and average SE) and ANOVA *p*-values for the fatty acid (FA) profile of cow and goat retail milk collected throughout the study.

	Cow	Goat		ANOVA
Individual FA and FA Groups	*n* = 48	*n* = 36	SE	*p*-Values *^a^*
**SFA (% of total FA) *^b^***				
C12:0	3.33	4.16	0.079	*
C14:0	11.1	10.3	0.09	**
C16:0	33.1	30.3	0.31	*
C18:0	9.95	9.08	0.152	ns
**MUFA (% of total FA) *^c^***				
OA	20.0	20.4	0.27	ns
VA	1.22	0.72	0.046	**
**PUFA (% of total FA) *^d^***				
LA	1.71	2.61	0.051	*
RA	0.591	0.469	0.0198	†
ALNA	0.439	0.342	0.0111	†
EPA	0.048	0.035	0.0012	*
DPA	0.079	0.075	0.0018	ns
DHA	0.007	0.015	0.0012	**
**FA groups (% of total FA)**				
SFA	68.8	70.3	0.29	*
MUFA	27.3	25.3	0.29	†
*cis* MUFA *^e^*	24.2	22.9	0.27	ns
*trans* MUFA *^f^*	3.10	2.45	0.054	***
PUFA	3.97	4.38	0.075	ns
*cis* PUFA *^g^*	2.59	3.36	0.056	*
*trans* PUFA *^h^*	0.034	0.006	0.0017	**
*cis/trans + trans/cis* PUFA *^i^*	1.34	1.02	0.032	**
*n*-3 *^j^*	0.792	0.538	0.0199	*
*n*-6 *^k^*	2.09	2.94	0.053	*
*trans* FA *^l^*	3.13	2.46	0.055	***
*trans* FA (exc. VA)	1.91	1.74	0.036	†
**Indices**				
*Human health-related*				
AI *^m^*	2.60	2.56	0.044	ns
TI *^n^*	3.13	3.13	0.042	ns
*n*-3/*n*-6	0.388	0.185	0.0105	**
*Δ^9^-desaturase activity*				
Δ^9^I *^o^*	0.297	0.304	0.0032	ns
C14:1/C14:0	0.084	0.015	0.0005	***
C16:1/C16:0	0.058	0.035	0.0005	***
OA/C18:0	2.01	2.27	0.026	†
RA/VA	0.491	0.678	0.0124	***

*^a^* Significances were declared at ***, *p* < 0.001; **, *p* < 0.01; *, *p* < 0.05; †, 0.05 < *p* < 0.10 (trend); ns, *p* > 0.10 (non-significant). *^b^* Saturated FA: C4:0, C5:0, C6:0, C7:0, C8:0, C9:0, C10:0, C11:0, C12:0, C13:0, C13:0*iso*, C13:0*anteiso*, C13:0, C14:0*iso*, C14:0, C15:0*anteiso*, C15:0, C16:0*iso*, C16:0, C17:0*iso*, C17:0, C18:0*iso*, C18:0, C20:0, C22:0, C24:0. *^c^* Monounsaturated FA (MUFA): *c*9 C10:1, *c*10 C11:1, *c*9 C12:1, *c*9 C13:1, *t*9 14:1, *c*9 C14:1, *c*10 C15:1, *t*7+t8 C16:1, *t*9 C16:1, *t*11+*t*12+*t*13 C16:1, *c*9 C16:1 (co-elutes with C17:0*anteiso*), *c*11 C16:1, *c*13 C16:1, *t*10 C17:1, *c*9 C17:1, *t*4 C18:1, *t*5 C18:1, *t*6+*t*7+*t*8 C18:1, *t*9 C18:1, *t*10 C18:1, *t*11 C18:1 (VA), *c*6+*t*12 C18:1, *c*9 C18:1 (OA), *t*15 C18:1, *c*11 C18:1, *c*12 C18:1, *c*13 C18:1, *t*16 + *c*14 C18:1, *c*15 C18:1 (co-elutes with C19:0), *c*16 C18:1, *c*5 C20:1, *c*8 C20:1, *c*11 C20:1, *c*13 C22:1, *c*15 C24:1. *^d^* Polyunsaturated FA (PUFA): *t*11*t*15 C18:2, *t*9*t*12 C18:2, *c*9*t*13 C18:2, *c*10*t*14 C18:2, *c*9*t*14 C18:2, *c*9*t*12 C18:2, *t*9*c*12 C18:2, *t*11*c*15 C18:2, *c*9*c*12 C18:2 (LA), *t*12*c*15 C18:2 (co-elutes with *c*9 C19:1), *c*6*c*9*c*12 C18:3, *c*9*c*12*c*15 C18:3 (ALNA), *c*9*c*11 C18:2 conjugated (RA) (co-elutes with *t*7*c*9+*t*8*c*10+*t*6*c*8 C18:2), other C18:2 conjugated FA of unknown isomerism, *c*11*c*14 C20:2, *c*8*c*11*c*14 C20:3, *c*11*c*14*c*17 C20:3, *c*5*c*8*c*11*c*14 C20:4, *c*13*c*16 C22:2, EPA, *c*13*c*16*c*19 C22:3, DPA, DHA. *^e^ cis* MUFA: *c*9 C10:1, *c*10 C11:1, *c*9 C12:1, *c*9 C13:1, *c*9 C14:1, *c*10 C15:1, *c*9 C16:1 (co-elutes with C17:0*anteiso*), *c*11 C16:1, *c*13 C16:1, *c*9 C17:1, *c*6 C18:1 (co-elutes with *t*12 C18:1), OA, *c*11 C18:1, *c*12 C18:1, *c*13 C18:1, *c*14 C18:1 (co-elutes with *t*16 C18:1), *c*15 C18:1 (co-elutes with C19:0), *c*16 C18:1, *c*5 C20:1, *c*8 C20:1, *c*11 C20:1, *c*13 C22:1, *c*15 C24:1. *^f^ trans* MUFA: *t*9 14:1, *t*7+t8 C16:1, *t*9 C16:1, *t*11+*t*12+*t*13 C16:1, *t*10 C17:1, *t*4 C18:1, *t*5 C18:1, *t*6+*t*7+*t*8 C18:1, *t*9 C18:1, *t*10 C18:1, VA, *t*12 C18:1 co-elutes with *c*6 C18:1), *t*15 C18:1, *t*16 C18:1 (co-elutes with *c*14 C18:1). *^g^ cis* PUFA: LA, ALNA, c11c14 C20:2, c8c11c14 C20:3, c11c14c17 C20:3, c5c8c11c14 C20:4, c13c16 C22:2, EPA, c13c16c19 C22:3, DPA, DHA. *^h^ trans* PUFA: *t*11*t*15 C18:2, *t*9*t*12 C18:2. *^i^ cis/trans* + *trans/cis* PUFA: *c*9*t*13 C18:2, *c*10*t*14 C18:2, *c*9*t*14 C18:2, *c*9*t*12 C18:2, *t*9*c*12 C18:2, *t*11*c*15 C18:2, *t*12*c*15 C18:2 (co-elutes with *c*9 C19:1), RA (co-elutes with *t*7*c*9+*t*8*c*10+*t*6*c*8 C18:2), other C18:2 conjugated FA of unknown isomerism. *^j^* omega-3 PUFA (*n*-3): *t*11*t*15 C18:2, *t*11*c*15 C18:2, *t*12*c*15 C18:2 (co-elutes with *c*9 C19:1), ALNA, *c*11*c*14*c*17 C20:3, EPA, *c*13*c*16*c*19 C22:3, DPA, DHA. *^k^* omega-6 PUFA (*n*-6): *t*9*t*12 C18:2, *c*9*t*12 C18:2, *t*9*c*12 C18:2, LA, *c*6*c*9*c*12 C18:3, *c*11*c*14 C20:2, *c*8*c*11*c*14 C20:3, *c*5*c*8*c*11*c*14 C20:4, *c*13*c*16 C22:2, *c*7*c*10*c*13*c*16 C22:4. *^l^ trans* FA: *t*9 C14:1, *t*9 C16:1, *t*11+*t*12+*t*13 C16:1, *t*10 C17:1, *t*4 C18:1, *t*5 C18:1, *t*6+*t*7+*t*8 C18:1, *t*9 C18:1, *t*10 C18:1, VA, *t*12 C18:1, *t*15 C18:1, *t*16 C18:1, *t*11*t*15 C18:2, *t*9*t*12 C18:2, *t*12*t*15 C18:2. *^m^* Atherogenicity index = (C12:0 + 4 x C14:0 + C16:0) / (MUFA + PUFA), as described in Srednicka-Tober et al. [43]. *^n^* Thrombogenicity index = (C14:0 + C16:0 + C18:0) / [(0.5 x MUFA) + (0.5 x *n*-6) + (3 x *n*-3) + (*n*-3/*n*-6)], as described in Srednicka-Tober et al. [43]. ^o^ Δ^9^-desaturase activity index = (c9 C14:1+c9 C16:1+OA+RA)/(c9 C14:1+c9 C16:1+OA+RA+C14:0+C16:0+C18:0+VA), as proposed by Kay et al. [36].

**Table 3:** Means (and average SE) and ANOVA *p*-values for the mineral concentrations of cow and goat retail milk collected throughout the study.

	Cow	Goat		ANOVA
Minerals	*n* = 47	*n* = 36	SE	*p*-Values *^a^*
As (μg/kg)	0.249	0.232	0.0237	ns
B (mg/kg)	0.176	0.263	0.0290	*
Ca (g/kg)	1.128	1.066	0.0084	**
Cd (μg/kg)	0.047	0.044	0.0088	ns
Co (μg/kg)	0.335	0.299	0.0339	ns
Cu (mg/kg)	0.035	0.070	0.0034	***
Fe (mg/kg)	0.214	0.216	0.0168	ns
I (mg/kg)	0.363	0.673	0.0346	*
K (g/kg)	1.528	2.037	0.0133	***
Mg (g/kg)	0.113	0.144	0.0013	***
Mn (mg/kg)	0.020	0.049	0.0010	***
Mo (mg/kg)	0.039	0.024	0.0023	ns
Na (g/kg)	0.377	0.354	0.0032	**
Ni (μg/kg)	1.151	0.826	0.2838	ns
P (g/kg)	0.908	0.986	0.0108	***
Pb (μg/kg)	0.583	0.374	0.1390	ns
S (mg/kg)	0.299	0.272	0.0040	*
Se (mg/kg)	0.016	0.017	0.0004	ns
Zn (mg/kg)	3.416	2.889	0.0413	**

*^a^* Significances were declared at ***, *p* < 0.001; **, *p* < 0.01; *, *p* < 0.05; †, 0.05 < *p* < 0.10 (trend); ns, *p* > 0.10 (non-significant).

**Table 4:** Means (and average SE) and ANOVA *p*-values for the phytoestrogen concentrations of cow and goat retail milk collected throughout the study.

	Cow	Goat		ANOVA
Phytoestrogens (ng/mL)	*n* = 48	*n* = 36	SE	*p*-Values *^a^*
**Plant lignans**				
Secoisolariciresinol	0.135	0.050	0.005	***
Matairesinol	0.123	0.061	0.008	**
Lariciresinol	0.343	0.180	0.010	***
Hydroxymatairesinol	0.176	0.077	0.009	***
Sum of plant lignans	0.777	0.369	0.023	***
**Mammalian lignans**				
Enterolactone	61.8	20.0	1.18	***
Enterodiol	0.334	0.726	0.037	†
Sum mammalian lignans	62.2	20.7	1.18	***
**Sum of lignans**	63.0	21.1	1.19	***
**Plant isoflavones**				
Daidzein	0.952	8.066	0.559	*
Genistein	0.833	9.350	1.154	†
Glycitein	2.07	5.52	0.139	***
Formononetin	0.082	6.479	0.770	ns
Naringenin	0.173	0.493	0.029	**
Sum of plant isoflavones	4.11	29.91	2.489	†
**Mammalian isoflavones**				
Equol	63.6	690.6	42.20	*
**Sum of isoflavones**	67.7	720.5	43.78	*
**Plant coumestans**				
Coumestrol	0.096	0.367	0.046	ns

*^a^* Significances were declared at ***, *p* < 0.001; **, *p* < 0.01; *, *p* < 0.05; †, 0.05 < *p* < 0.10 (trend); ns, *p* > 0.10 (non-significant).

## Supplementary Materials

The following are available online at https://www.mdpi.com/2072-6643/11/10/2282/s1, Table S1: Means (and average SE) and ANOVA *p*-values for the concentrations of all individual fatty acids of cow and goat retail milk collected, which were quantified in the study, Figure S1: Interaction means ± SE (error bars) for the effects of species (cow, goat) and month (in order of appearance from left to right in Axis X: M, March; A, April; M, May; J, June; J, July; A, August; S, September; O, October; N, November; D, December; J, January; F, February) on the health indices of retail milk: (I) AI, atherogenicity index [43]; (II) TI, thrombogenicity index [43]; (III) *n*-3/*n*-6, ratio of omega-3 to omega-6 fatty acids. P represents the ANOVA *p*-value for the interaction. Means for species and within a month with different lowercase letters are significantly different according to Fisher’s Least Significant Difference test (*p* < 0.05), Figure S2: Interaction means ± SE (error bars) for the effects of species (cow, goat) and month (in order of appearance from left to right in Axis X: M, March; A, April; M, May; J, June; J, July; A, August; S, September; O, October; N, November; D, December; J, January; F, February) on the Δ^9^-desaturase activity indices (I) Δ^9^I was calculated as shown in Kay et al. [36]; (II) ratio of c9 C14:1/C14:0; (III) ratio of c9 C16:1/C16:0; (IV) ratio of c9 C18:1 (OA, oleic acid)/C18:0; (V) ratio of c9t11 C18:2 (RA, rumenic acid)/t11 C18:1 (VA, vaccenic acid) P represents the ANOVA *p*-value for the interaction. Means for species and within a month with different lowercase letters are significantly different according to Fisher’s Least Significant Difference test (*p* < 0.05).
