# Supplementary material for: Comparative Nutrient Profiling of Retail Goat and Cow Milk
Source: Nutrients. 2019 Sep 24;11(10):2282. doi: 10.3390/nu11102282 (PMC6835441; doi:10.3390/nu11102282)
Supplement: Supplementary file 1 [file nutrients-11-02282-s001.pdf]

SUPPLEMENTARY MATERIAL

Table S1. Means (and average SE) and ANOVA *p*-values for the concentrations of all individual fatty acids of cow and goat retail milk collected, which were quantified in the study

Fatty acids (% of total)	Cow *n* = 48	Goat *n* = 36	SE	ANOVA *p*-values
C4:0	2.07	1.42	0.043	**<0.001**
Unknown	0.017	0.013	0.0009	0.167
C5:0	0.021	0.011	0.0004	**<0.001**
C6:0	1.53	1.70	0.036	**<0.001**
C7:0	0.022	0.023	0.0008	0.473
C8:0	1.00	2.07	0.033	**<0.001**
C9:0	0.029	0.049	0.0011	**<0.001**
C10:0	2.51	8.00	0.090	**<0.001**
c9 C10:1	0.244	0.184	0.0050	**<0.001**
C11:0	0.055	0.075	0.0019	**0.005**
C12:0	3.33	4.16	0.079	**0.012**
C13:0 iso	0.027	0.015	0.0006	**0.001**
C13:0 anteiso	0.015	0.008	0.0015	**0.009**
c9 C12:1	0.083	0.081	0.0021	0.742
C13:0	0.091	0.068	0.0015	**0.002**
C14:0 iso	0.081	0.076	0.0011	0.102
C14:0	11.1	10.3	0.09	**0.001**
t9 C14:1	0.216	0.166	0.0027	**0.001**
C15:0 anteiso	0.423	0.283	0.0072	**<0.001**
c9 C14:1	0.931	0.157	0.0074	**<0.001**
C15:0	1.03	0.73	0.010	**<0.001**
C16:0 iso	0.199	0.225	0.0023	**0.014**
C16:0	33.1	30.3	0.31	**0.017**
t6+t7+t8 C16:1	0.032	0.044	0.0013	**0.003**
t9 C16:1	0.009	0.016	0.0006	**0.014**
C17:0 iso	0.384	0.374	0.0066	0.621
t11+t12+t13 C16:1	0.196	0.301	0.0036	**<0.001**
c9 C16:1 + C17 anteiso	1.91	1.05	0.013	**<0.001**
c11 C16:1	0.143	0.019	0.0030	**<0.001**
c13 C16:1	0.140	0.191	0.0066	**0.004**
C17:0	0.487	0.460	0.0085	0.144
t10 c17:1	0.050	0.062	0.0022	**<0.001**
C18:0 iso	0.057	0.027	0.0015	**0.002**
c9 C17:1	0.211	0.221	0.0033	0.230
C18:0	9.95	9.08	0.152	0.135
t4 C18:1	0.016	0.009	0.0006	**0.002**
t5 C18:1	0.011	0.009	0.0004	0.119
t6+t7+t8 C18:1	0.276	0.223	0.0054	**0.032**
t9 C18:1	0.193	0.218	0.0058	***0.095***
t10 C18:1	0.370	0.339	0.0218	0.532
t11 C18:1	1.22	0.72	0.046	**0.004**
c6 + t12 C18:1	0.283	0.193	0.0105	**0.002**
c9 C18:1	20.0	20.4	0.27	0.662
t15 C18:1	0.208	0.140	0.0113	**<0.001**
c11 C18:1	0.589	0.478	0.0140	0.169
c12 C18:1	0.224	0.217	0.0067	0.778
c13 C18:1	0.091	0.055	0.0013	**<0.001**
t16 + c14 C18:1	0.325	0.223	0.0050	**<0.001**
c15 C18:1 + C19:0	0.127	0.088	0.0051	**<0.001**
t11t15 C18:2	0.027	0.003	0.0013	**0.002**
t9t12 C18:2	0.007	0.003	0.0005	**<0.001**

c9t13 C18:2	0.210	0.210	0.0052	0.991
c10t14 C18:2	0.110	0.047	0.0019	**<0.001**
c9t14 C18:2	0.125	0.145	0.0024	**0.015**
c9t12 C18:2	0.062	0.048	0.0010	**0.002**
c16 C18:1	0.034	0.028	0.0009	**0.044**
t11c15 C18:2	0.150	0.040	0.0060	**0.002**
t9c12 C18:2	0.019	0.009	0.0011	**0.002**
c9c12 C18:2	1.71	2.61	0.051	**0.015**
t12c15 C18:2 + c9 C19:1	0.043	0.028	0.0045	**0.014**
C20:0	0.140	0.178	0.0030	**0.002**
c6c9c12 C18:3	0.026	0.030	0.0009	0.146
c8 C20:1	0.102	0.021	0.0010	**<0.001**
c11 C20:1	0.039	0.045	0.0037	**0.024**
c9c12c15 C18:3	0.439	0.342	0.0111	***0.050***
c9t11 C18:2	0.591	0.469	0.0198	***0.070***
Unknown C18:2 conjugated	0.029	0.028	0.0022	0.676
Unknown C18:2 conjugated	0.027	0.013	0.0014	**0.006**
c11c14 C20:2	0.020	0.017	0.0010	0.206
C22:0	0.056	0.039	0.0011	**0.002**
c8c11c14 C20:3	0.081	0.024	0.0009	**<0.001**
c13 C22:1	0.016	0.010	0.0014	0.168
c11c14c17 C20:3	0.010	0.007	0.0008	**0.015**
c5c8c11c14 C20:4	0.106	0.159	0.0022	**<0.001**
c13c16 C22:2	0.040	0.019	0.0011	**0.002**
c5c8c11c14c17 C20:5	0.048	0.035	0.0012	**0.017**
C24:0	0.035	0.013	0.0006	**<0.001**
c13c16c19 C22:3	0.010	0.007	0.0007	**<0.001**
c7c10c13c16 C22:4	0.017	0.017	0.0014	0.888
c7c10c13c16c19 C22:5	0.079	0.075	0.0018	0.439
c4c7c10c13c16c19 C22:6	0.007	0.015	0.0012	**0.004**

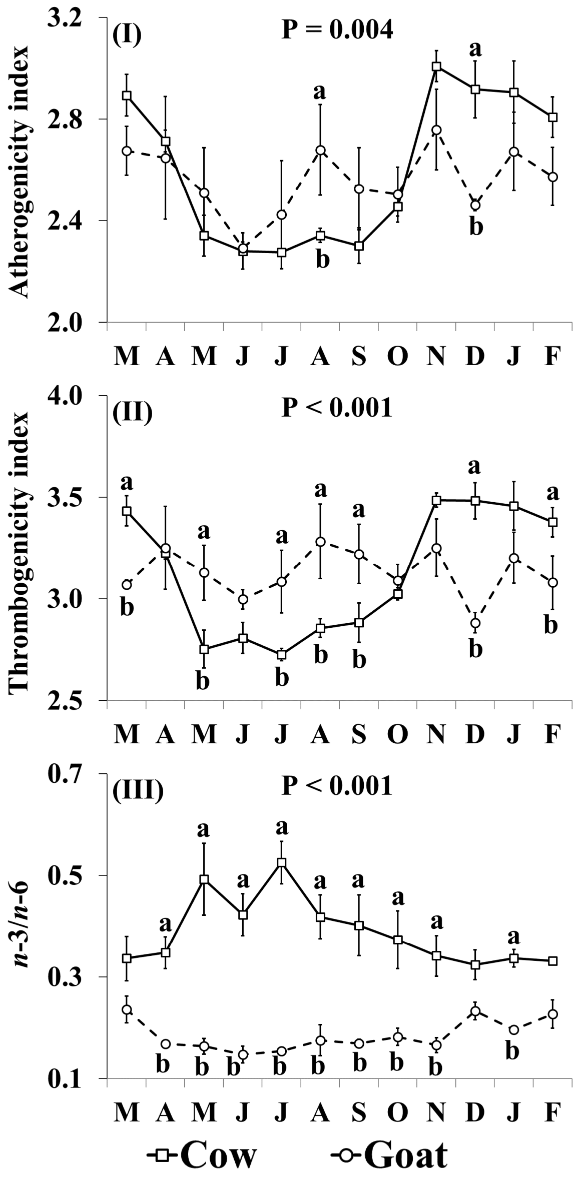

Figure S1. Interaction means ± SE (error bars) for the effects of species (cow, goat) and month (in order of appearance from left to right in Axis X: M, March; A, April; M, May; J, June; J, July; A, August; S, September; O, October; N, November; D, December; J, January; F, February) on the health indices of retail milk: (I) AI, atherogenicity index [43]; (II) TI, thrombogenicity index [43]; (III) *n*-3/*n*-6, ratio of omega-3 to omega-6 fatty acids. P represents the ANOVA *p*-value for the interaction. Means for species and within a month with different lower case letters are significantly different according to Fisher's Least Significant Difference test ($p < 0.05$).

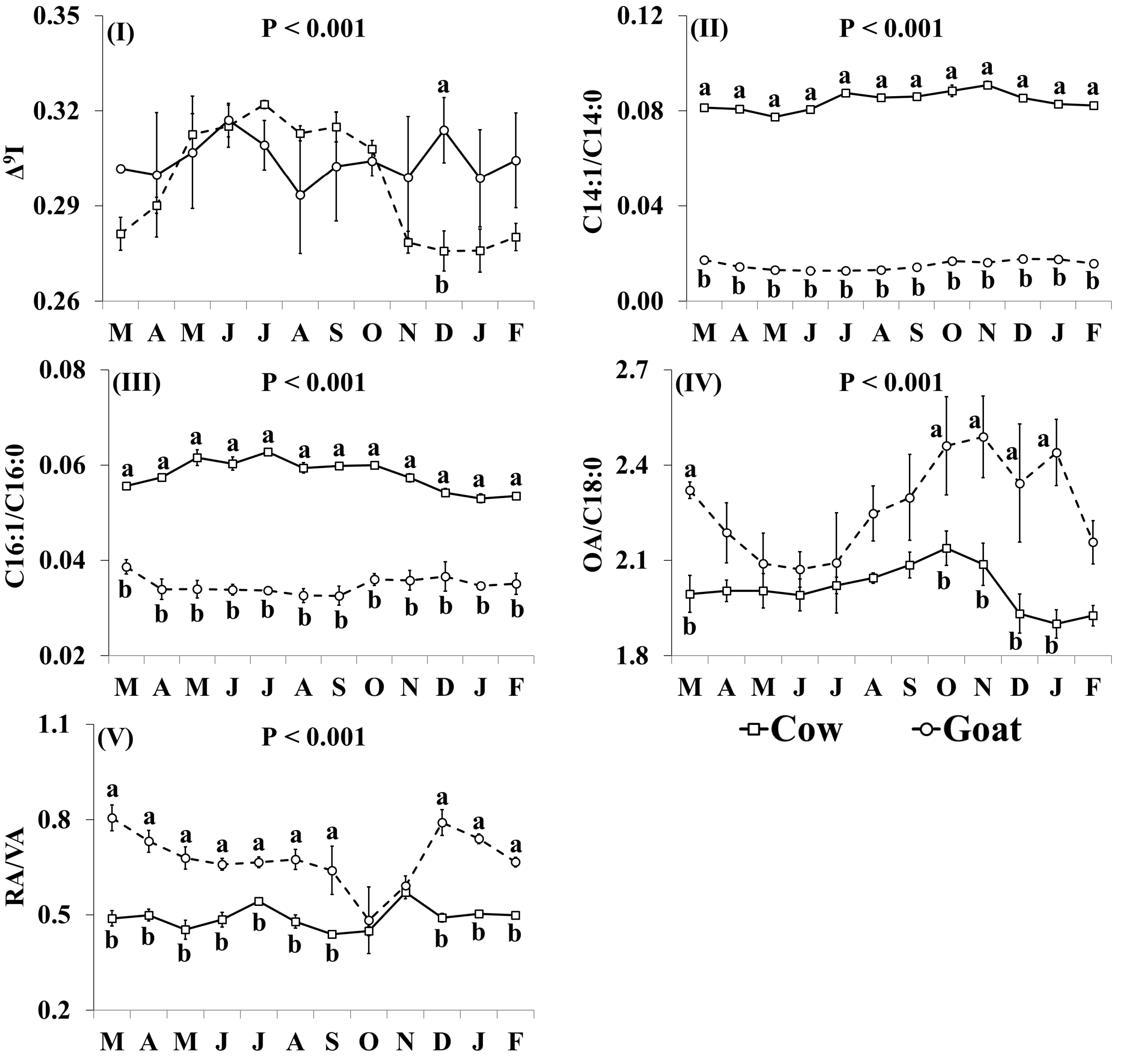

Figure S2. Interaction means ± SE (error bars) for the effects of species (cow, goat) and month (in order of appearance from left to right in Axis X: M, March; A, April; M, May; J, June; J, July; A, August; S, September; O, October; N, November; D, December; J, January; F, February) on the Δ^9-desaturase activity indices: (I) Δ^9I was calculated as shown in Kay et al. [36]; (II) ratio of c9 C14:1/C14:0; (III) ratio of c9 C16:1/C16:0; (IV) ratio of c9 C18:1 (OA, oleic acid)/C18:0; (V) ratio of c9t11 C18:2 (RA, rumenic acid)/t11 C18:1 (VA, vaccenic acid) P represents the ANOVA *p*-value for the interaction. Means for species and within a month with different lower case letters are significantly different according to Fisher's Least Significant Difference test ($p < 0.05$).
